# A Cross-Sectional Study of HPV Vaccine Acceptability in Gaborone, Botswana

**DOI:** 10.1371/journal.pone.0025481

**Published:** 2011-10-25

**Authors:** Yumi Taylor DiAngi, Catherine A. Panozzo, Doreen Ramogola-Masire, Andrew P. Steenhoff, Noel T. Brewer

**Affiliations:** 1 Department of Internal Medicine, University of Pennsylvania, Philadelphia, Pennsylvania, United States of America; 2 Botswana-University of Pennsylvania Partnership, Gaborone, Botswana; 3 Department of Epidemiology, Gillings School of Global Public Health, University of North Carolina, Chapel Hill, North Carolina, United States of America; 4 Department of Obstetrics and Gynecology, University of Pennsylvania, Philadelphia, Pennsylvania, United States of America; 5 Departments of Internal Medicine and Obstetrics and Gynecology, University of Botswana, Gaborone, Botswana; 6 Department of Pediatrics, University of Pennsylvania, Philadelphia, Pennsylvania, United States of America; 7 Department of Pediatrics, The Children's Hospital of Philadelphia, Philadelphia, Pennsylvania, United States of America; 8 Health Behavior and Health Education, Gillings School of Global Public Health, University of North Carolina, Chapel Hill, North Carolina, United States of America; 9 Lineberger Comprehensive Cancer Center, University of North Carolina, Chapel Hill, North Carolina, United States of America; Instituto de Pesquisa Clínica Evandro Chagas/Fundação Oswaldo Cruz, Brazil

## Abstract

**Background:**

Cervical cancer is the most common cancer among women in Botswana and elsewhere in Sub-Saharan Africa. We sought to examine whether HPV vaccine is acceptable among parents in Botswana, which recently licensed the vaccine to prevent cervical cancer.

**Methods and Findings:**

We conducted a cross-sectional survey in 2009, around the time the vaccine was first licensed, with adults recruited in general medicine and HIV clinics in Gaborone, the capital of Botswana. Although only 9% (32/376) of respondents had heard of HPV vaccine prior to the survey, 88% (329/376) said they definitely will have their adolescent daughters receive HPV vaccine. Most respondents would get the vaccine for their daughters at a public or community clinic (42%) or a gynecology or obstetrician's office (39%), and 74% would get it for a daughter if it were available at her school. Respondents were more likely to say that they definitely will get HPV vaccine for their daughters if they had less education (OR = 0.20, 95% CI = 0.07–0.58) or lived more than 30 kilometers from the capital, Gaborone (OR = 2.29, 95% CI = 1.06–4.93). Other correlates of acceptability were expecting to be involved in the decision to get HPV vaccine, thinking the vaccine would be hard to obtain, and perceiving greater severity of HPV-related diseases.

**Conclusions:**

HPV vaccination of adolescent girls would be highly acceptable if the vaccine became widely available to the daughters of healthcare seeking parents in Gaborone, Botswana. Potential HPV vaccination campaigns should provide more information about HPV and the vaccine as well as work to minimize barriers.

## Introduction

About 530,000 women are diagnosed with cervical cancer every year worldwide, and about 275,000 women die from the disease [1]. About 15% (80,000) of these diagnoses and 20% (53,000) of these deaths occur in Africa, where cervical cancer screening and treatment is much more limited than in the developed world [1]. Another important contributor to the cervical cancer burden in Africa is the high prevalence of HIV, especially in Sub-Saharan Africa (5%) [2]. Women with HIV are six times more likely to develop cervical cancer compared to those not infected with HIV, due to HIV-related immune suppression [3]. Many countries have approved vaccines to prevent infection with human papillomavirus (HPV), several strains of which can cause cervical cancer [4]. In lower resource settings where cervical cancer screening and treatment are scarce, delivery of the 3-dose HPV vaccine series could provide women with ongoing protection, if it became widely available at a low cost (e.g., if the Global Alliance for Vaccines and Immunizations negotiates lower prices for the vaccine in Botswana).

Early research in the US, Canada, Europe and Australia found that correlates of HPV vaccine acceptability included the belief that cervical cancer is a common and serious health threat, that the vaccine is safe, effective and easy to obtain, and that a doctor would recommend it, all constructs from the health belief model [5]. More recent work has focused on HPV vaccine acceptability in developing country settings such as Kenya, Latin America, the Caribbean, Vietnam, Malaysia, China, and India [6]–[12]. While these studies have generally found high acceptability of HPV vaccine, few studies have quantitatively examined the acceptability of HPV vaccine in Sub-Saharan Africa [6], [13], [14], and these studies had limited information on respondents' beliefs about the vaccine.

Botswana, a middle income Sub-Saharan African country, has a limited cervical cancer screening program and high HIV burden, both factors that contribute to high mortality from cervical cancer [2], [15], [16]. The Botswana government licensed HPV vaccine in 2009 but, likely due to its relatively high cost, the vaccine is not yet available in the public sector, where the majority of the population accesses care. When HPV vaccine becomes available, the national health plan may one day cover the vaccine, and it may also be available sooner to some Botswana parents if they paid for it out of pocket. The purpose of the present study was to examine HPV vaccine acceptability for adolescent girls and its predictors among healthcare-seeking adults in Gaborone, the capital of Botswana. Based on the health belief model, we expected higher acceptability of HPV vaccine among respondents who perceived cervical cancer to be likely and severe, thought the vaccine would not be hard to obtain, and would be recommended by a doctor.

## Methods

### Ethics Statement

The Institutional Review Boards at the University of Pennsylvania, Princess Marina Hospital, and the Botswana Ministry of Health approved this study and consent process for this study. We obtained written and verbal consent from all participants. Completion of the survey was considered documentation of verbal consent. Participants received no monetary, food, or other incentives for participating to minimize the possibility of coercion.

### Participants and Procedures

We conducted a cross-sectional survey of parents' and adults' intentions to get HPV vaccine for adolescent daughters in Gaborone, Botswana. In January 2009, we identified potential participants from two free health clinics at Princess Marina Hospital, the capital's main government hospital. We recruited in clinics associated with an existing Botswana-University of Pennsylvania partnership due to limited resources. Both clinics provided virtually free health care to citizens of Botswana and charge a subsidised fee to non-citizens. Since January 2002, Botswana has provided free highly active antiretroviral therapy (HAART) to citizens who are HIV positive and who meet specified clinical criteria.

Local Botswana high school graduates conducted interviews as part of a capacity building collaboration with the Botswana government. Interviewers received training about cervical cancer and appropriate translations for medical terminology from a Motswana obstetrics and gynecology physician. They memorized scripts to achieve consistency during introductions, interviews, and informed consent and received ongoing supervision throughout the interview month. The three interviewers rotated among study sites, and three experienced research nurses affiliated with the Botswana-University of Pennsylvania Partnership provided supervision.

In the study clinics, patients queued up in a large general seating area on a first come, first served basis. Each morning, a charge nurse provided general clinic information to these patients. Interviewers introduced the study in both English and Setswana (a local language), describing the survey as being about individual attitudes toward cervical cancer and new treatment options that could affect adolescent girls. Interviewers asked patients interested in volunteering to raise their hands and later obtained verbal consent. Due to space constraints and concerns about participants losing their spot in line, participants completed the survey in the main waiting area.

We offered the five-page, self-administered paper and pencil survey in either Setswana or English. Interviewers read each survey question for the few individuals that self-identified as unable to read or who preferred this option. Each day, interviewers conducted a head count at each study site to estimate what percentage of the population agreed to participate. We determined sample size based on our ability to recruit for one month in clinics in Gaborone as part of a capacity building exercise. An estimated half of people attending the clinics on the days of the study participated in the survey.

### Measures

The surveys appear in the appendix (Text S1). We modified survey items that we previously used in our HPV vaccine research with US adolescent females, parents, and healthcare providers [17]–[20]. We drafted our survey in English. A native Botswana resident and a Motswana obstetrics and gynecology physician translated the survey to Setswana and back translated it; we resolved discrepancies through discussion.

Since many pilot participants knew little about HPV, HPV vaccine, and cervical cancer, the survey provided basic information on these topics. For example, the information on the vaccine that appeared directly before the relevant HPV vaccine questions read, “An HPV vaccine is now available that protects against most genital warts and cervical cancer. Sometimes it's called the cervical cancer vaccine, HPV shot, Cervarix or Gardasil. We will call it the HPV vaccine.” We tested the survey's cultural appropriateness and clarity with three Botswana women and made several revisions to it.

The main outcome was willingness to vaccinate, assessed by a question that asked how likely adults were to get HPV vaccine for an adolescent daughter when it became available in Botswana. The response options were “definitely will not,” “probably will not,” “probably will,” and “definitely will”. We classified each participant as either definitely will get her or his daughter HPV vaccine ( = 1) or other responses ( = 0). Instructions stated that respondents who did not have an adolescent daughter should answer the survey questions as if they did.

### Statistical Methods

We use bivariate logistic regression models to examine bivariate correlates of HPV vaccine acceptability. We report bivariate analyses as exploratory multivariate models were unstable, perhaps due to limited variability in the outcome measure and our relatively small sample size. Less than 5% of data was missing for any variable. We analyzed data using SPSS version 16.0 (SPSS Inc., Chicago IL) and SAS version 9.2 (Cary, NC). All statistical tests were two-tailed, using a critical alpha of 0.05.

## Results

Most (77%) of the 376 survey respondents were female (Table 1). Their median age was 37 years (range 20 to 84 years). Respondents were distributed in similar proportions among three education levels: 31% had completed a primary education (similar to completing middle school or completing grade 6 in the US) or had less education, 41% secondary education (similar to completing high school in the US), and 28% tertiary education and above. Most respondents had no regular income (48%) or made less than US$360 (2500 pula) per month (29%), an amount considered low income by Botswana federal government tax brackets [21]. About two-thirds (65%) reported living within 30 kilometers of the capital, Gaborone. Most respondents had children (83%), and of these parents, 77% had one or more daughters. The median age of the 316 daughters was 17 years (range, 10 months to 42 years). Of the 241 respondents who had daughters, 71% had at least one daughter who was between the ages of 11 and 26, meaning that she was in the age range recommended to receive HPV vaccine.

**Table 1:** Demographic characteristics (correlates of HPV vaccine acceptability for daughters).

	No. definitely will get HPV vaccine for daughter/total no. in category (%)	Bivariate OR (95% CI)
Age		
20–30 years	102/115 (89)	ref.
31–84 years	226/257 (88)	0.93 (0.47–1.85)
Sex		
Female	252/287 (88)	ref.
Male	77/86 (90)	1.19 (0.55–2.58)
Marital Status		
Never married	214/243 (88)	ref.
Married/was married	114/129 (88)	1.03 (0.53–2.00)
Education		
Primary or less	111/115 (97)	ref.
More than primary	218/257 (85)	0.20 (0.07–0.58)*
Monthly income		
No regular income	157/177 (89)	ref.
<US$360 (2500 pula)	100/109 (92)	1.42 (0.62–3.23)
≥US$360 (2500 pula)	69/84 (82)	0.59 (0.28–1.21)
Distance from capital (Gaborone)		
0–29 km	202/237 (85)	ref.
≥30 km	119/128 (93)	2.29 (1.06–4.93)*
Children		
No	54/64 (89)	ref.
Yes, but no daughter	61/68 (90)	1.07 (0.35–3.24)
Yes, at least one daughter	211/241 (88)	0.86 (0.36–2.07)
Daughter in age range recommended to receive HPV vaccine		
No	118/132 (89)	ref.
Daughter outside of age range	61/70 (87)	0.80 (0.33–1.96)
Daughter ages 11 to 26	150/171 (88)	0.85 (0.41–1.74)
Study Site		
HIV clinic	151/170 (89)	ref.
General medicine clinic	178/203 (88)	0.90 (0.46–1.69)

*Note*. Counts do not add up to *n* = 376 for some variables due to missing data. HPV = human papillomavirus, OR = odds ratio, CI = confidence interval, ref. = referent group, HIV = human immunodeficiency virus.

*p<0.05.

About half (52%) of the respondents reported that they were HIV-positive (Table 2). Some respondents reported a history of sexually transmitted infections (23%) or genital warts (8%). Of the 289 female respondents, 29% reported never having had a previous Pap test, and 10% reported a previous diagnosis of cervical cancer. About 45% of the respondents completed the survey at the HIV clinic; nearly all (94%) of the respondents in the HIV clinic reported that they were HIV-positive. HIV clinic respondents were more likely than general medicine clinic respondents to be HIV-positive, older, female, or less educated, or to have had a sexually transmitted infection or genital warts.

**Table 2:** Personal health (correlates of HPV vaccine acceptability for daughters).

	No. definitely will get HPV vaccine for daughter/total no. in category (%)	Bivariate OR (95% CI)
Had sexually transmitted infection		
No	247/284 (87)	ref.
Yes	77/84 (92)	1.65 (0.71–3.84)
Had genital warts		
No	285/325 (88)	ref.
Yes	28/31 (90)	1.31 (0.38–4.51)
Had cervical cancer		
No	238/273 (87)	ref.
Yes	29/30 (97)	4.27 (0.56–32.30)
HIV status		
Negative (or never tested)	155/179 (87)	ref.
Positive	173/193 (90)	1.34 (0.71–2.52)

*Note*. Counts do not add up to *n* = 376 for some variables due to missing data. HPV = human papillomavirus, OR = odds ratio, CI = confidence interval, ref. = referent group, HIV = human immunodeficiency virus.

*p<0.05.

### Cervical Cancer and HPV Vaccine

Many respondents had heard of cervical cancer (71%) or genital warts (65%) prior to the survey. However, only about one-third had heard of HPV (35%). The majority thought their daughters had a moderate to high chance of getting genital warts (68%), HPV (72%), or cervical cancer (78%) in their lifetime (Table 3). About three-fourths rated these outcomes as severe or extremely severe (72%, 75%, and 78%).

**Table 3:** Beliefs about HPV-related diseases.

	No. definitely will get HPV vaccine for daughter/total no. in category (%)	Bivariate OR (95% CI)
Chance daughter will get HPV		
Less than high	205/237 (87)	ref.
High	121/133 (91)	1.57 (0.78–3.17)
Chance daughter will get genital warts		
No, low or moderate	212/237 (89)	ref.
High	107/123 (87)	1.23 (0.62–2.42)
Chance daughter will get cervical cancer		
No, low or moderate	183/209 (88)	ref.
High	142/160 (89)	1.12 (0.59–2.12)
Perceived severity of HPV infection for daughter		
Less than high	77/91 (85)	ref.
High	248/278 (89)	1.50 (0.76–2.98)
Perceived severity of genital warts for daughter		
Less than high	86/104 (83)	ref.
High or extremely high	240/265 (91)	2.01 (1.04–3.86)*
Perceived severity of cervical cancer for daughter		
Less than high	64/80 (80)	ref.
High or extremely high	260/288 (90)	2.32 (1.19–4.55)*

*Note*. Counts do not add up to *n* = 376 for some variables due to missing data. HPV = human papillomavirus, OR = odds ratio, CI = confidence interval, ref. = referent group.

*p<0.05.

Prior to the survey, few respondents had heard of HPV vaccine (9%), but most wanted more information about it (75%) (Table 4). The majority reported that they would be involved a lot in the decision to give HPV vaccine to their daughters (81%). Others involved in the decision would be her doctor (58%), the parent's spouse or partner (45%), the daughter (34%), an elder (19%), or other people (4%). Among respondents who said they would be involved in the decision to vaccinate their daughters, almost all (99%) said they would discuss the decision with them.

**Table 4:** Beliefs about HPV vaccine (correlates of HPV vaccine acceptability for daughters).

	No. definitely will get HPV vaccine for daughter/total no. in category (%)	Bivariate OR (95% CI)
Wanted more information about HPV vaccine		
Disagree	76/91 (84)	ref.
Agree	251/279 (90)	1.77 (0.90–3.48)
Would be involved in decision to give HPV vaccine		
No	57/71 (80)	ref.
Yes	271/301 (90)	2.22 (1.11–4.45)*
Perception of difficulty obtaining HPV vaccine		
Not hard	250/277 (90)	ref.
Hard	75/91 (82)	0.51 (.26–.99)*
Believed HPV vaccine increases likelihood of sex		
No	253/286 (88)	ref.
Yes	72/82 (88)	0.93 (0.44–2.00)

*Note*. Counts do not add up to *n* = 376 for some variables due to missing data. HPV = human papillomavirus, OR = odds ratio, CI = confidence interval, ref. = referent group.

*p<0.05.

Eighty-eight percent of respondents said they would definitely get HPV vaccine for their adolescent daughters. Fewer said they would definitely get it if a nurse recommended it than if a doctor did (78% vs. 89%, *p*<.001). Eighty-two percent would definitely give HPV vaccine to their daughters if it were available with other childhood vaccines. Twenty-two percent believed their daughters would be more likely or much more likely to have sex if they got HPV vaccine. The median range that respondents said they would be willing to pay for HPV vaccine was 21–50 pula (US$3.07-US$7.30).

The most common place people would get the vaccine for their daughters was a public or community clinic (42%), followed by a gynecology or obstetrician's office (39%). If HPV vaccine were available at their daughters' schools, 74% would get it for them there. Only 8% thought it would be hard or very hard to find a clinic with HPV vaccine. The most common barriers that respondents listed for getting HPV vaccine were “don't know” (59%), difficulties accessing a clinic, healthcare provider, or the vaccine (12%) and lack of information (10%).

### Correlates of HPV Vaccine Acceptability

Respondents with more than a primary education were less likely to say they would definitely get HPV vaccine for their daughters than those with a primary education or less (OR = 0.20, 95% CI = 0.07–0.58) (See Tables 2, 3, 4). Those living more than 30 kilometers from the capital were more willing than respondents living closer to or in the capital, Gaborone (OR = 2.29, 95% CI = 1.06 to 4.93) and those responding that they would be involved in the decision to get HPV vaccine were also more willing than those not involved in the decision (OR = 2.22, 95% CI = 1.11 to 4.45). Participants that thought HPV vaccine would be hard to obtain were less likely to say they will definitely get it for their daughters compared to those that thought the vaccine would be easy to obtain (OR = .51, 95% CI = .26 to .99). Finally, those that perceived genital wart or cervical cancer as severe or very severe compared to those that perceived such conditions as less than severe were more willing to get HPV vaccine for their daughters (OR = 2.01, 95% CI = 1.04 to 3.86 and OR = 2.32, 95% CI = 1.19 to 4.55).

## Discussion

HPV vaccine acceptability was high among respondents in this study of adults in Botswana, a middle-income African country with a high rate of cervical cancer. The high acceptability is typical of studies of acceptability of providing HPV vaccine to daughters [5] [22], though it is somewhat counterintuitive given respondents' low awareness of HPV and HPV vaccine prior to the study. Other studies on HPV vaccine acceptability have shown similar findings. Studies in Kisumu, Kenya and Mysore, India found that despite limited knowledge about HPV vaccine, most parents were highly accepting of having their daughters receive the vaccine [6], [11].

Our findings suggest an important role for providing information about the disease and interventions available to help respondents and their daughters make informed decisions about whether to get them HPV vaccine. Almost three-quarters of survey participants wanted more information about HPV vaccine. Furthermore, almost all respondents who would be involved in the decision to vaccinate their daughters would discuss the decision with her.

Efforts to spread information may wish to consider addressing several health belief model constructs that were associated with acceptability [18], [23] Our respondents reported higher acceptability if they believed two HPV-related conditions, genital warts and cervical cancer, were serious health problems (i.e., perceived severity), but beliefs about the chances of their daughter getting these illnesses were not associated with interest in the vaccine (i.e., perceived likelihood). Acceptability was lower if they believed the vaccine would be hard to obtain for their daughters, but potential sexual disinhibition was not a concern (i.e., perceived barriers). A recommendation from doctors was a more potent cue to action than a recommendation from a nurse (i.e., cue to action).

Although the majority of respondents did not know what barriers might prevent them from getting HPV vaccine, our study revealed some possibilities. Given that the median amount that participants were willing to pay for HPV vaccine was about US$5, cost would likely be a major barrier if HPV vaccine was not heavily subsidized or offered for free by the government. Furthermore, although about 80% of participants said they would get HPV vaccine at a clinic or gynecology office, nearly as many were willing to get the vaccine at their daughters' schools. Given these findings and that HPV school-based vaccine clinics have been successful elsewhere [24], [25], programs should consider schools as potential venues for HPV vaccine delivery in Botswana. Schools have already been an effective delivery site for other vaccines, and they may help families who have difficulties accessing a clinic or healthcare provider to obtain HPV vaccine for their daughters.

The association of higher education levels with lower willingness to vaccinate has also been shown in the United States [26], but this is the first replication to our knowledge of this finding for HPV vaccine in a developing country. The finding that participants living further from the capital, typically in more rural areas, were more willing to vaccinate their daughters than those living in the urban capital has not been described previously in other countries to our knowledge. The lower access to resources generally in rural areas, and the consequences of having to travel for medical care, may account for this higher interest.

### Limitations

Study limitations include that the convenience sample of adults seeking healthcare may not be representative of Botswana. These individuals may have had different awareness of and access to healthcare than the general population. Although we oversampled HIV-positive persons (52% in our study vs. 24% national prevalence in adults 15–49), results were similar when stratified by HIV status (data not shown), suggesting that this oversampling did not materially affect our results. Though HIV status was self-reported, HIV prevalence in the general medicine clinic was reasonably close to the national prevalence (17% vs. 24%), providing some confidence that patients reported their HIV status with reasonable accuracy. While some respondents did not have daughters, we found no differences in HPV vaccine acceptability among respondents with daughters compared to those with only sons or no children.

Our survey results suggest that HPV vaccine would be an accepted addition to routine care in Botswana that could play a central role in cervical cancer prevention, but future studies should also strive to include non-healthcare seeking individuals in both rural and urban settings in Botswana and then replicate such studies throughout sub-Saharan Africa. These studies should also seek to determine the prevalent strains of HPV to determine what percentage of those infected are at risk of developing cervical cancer. Widespread uptake of the vaccine could help to mitigate cervical cancer risk posed by very low rates of Pap testing and condom use [2], [27]. However, the feasibility of such action depends in part upon the affordability of HPV vaccine which at the time of our study was priced at US$360 for three doses, more than the monthly salary of three-quarters of our survey respondents [28]. The Global Alliance for Vaccines and Immunizations (GAVI) is currently negotiating lower prices for HPV vaccine in developing countries. If GAVI is successful and Botswana qualified for GAVI pricing, Botswana could adopt HPV vaccine into its multi-year plan for immunization and make it widely available to women. Future studies should perform comparative analyses of the costs of HPV vaccination versus traditional cervical cancer screening methods to help national programs in Botswana and elsewhere determine their relative benefits. As Botswana has such a high incidence of cervical cancer and cervical cancer has the highest mortality rate of all cancers in Botswana, a successful HPV vaccination program could significantly reduce cervical cancer morbidity and mortality [15].

## Supporting Information

Text S1 **Gaborone Children's Health Study Questionnaire in English and Setswana.**
(PDF)
