# Supplementary material for: A Cross-Sectional Study of HPV Vaccine Acceptability in Gaborone, Botswana
Source: PLoS One. 2011 Oct 25;6(10):e25481. doi: 10.1371/journal.pone.0025481 (PMC3201944; doi:10.1371/journal.pone.0025481)
Supplement: Text S1 — Gaborone Children's Health Study Questionnaire in English and Setswana. (PDF) [file pone.0025481.s001.pdf]

Gaborone Children's Health Study [English version of survey, Setswana translation appears at end of document]

Thank you for your help on this important study which is an international collaborative effort. This survey is about several health topics important to women and their families. Remember, there are no right or wrong answers to these questions. We would like to know what ***you*** think about these important topics.

A. General

	Yes	No	
A1. Have you ever heard of human papillomavirus or HPV? HPV is different from **HIV**.	☐	☐	
A2. Have you ever heard of genital warts?	☐	☐	
A3. Have you ever heard of cancer of the cervix or cervical cancer?	☐	☐	If **no**, skip to question **B.**

A4. If a woman had cervical cancer, list three things *you think* are most likely to have caused it.

#1 (most likely)____

#2 ____

#3____

The next questions are about **HIV** (human immunodeficiency virus) and cervical cancer.

	Lowers her chance	No effect	Raises her chance	Don't Know
A5. How do you think **HIV** affects a woman's chance of getting cervical cancer?	☐	☐	☐	☐
A6. How do you think **HIV** affects a woman's chance of getting HPV infection?	☐	☐	☐	☐

B. Daughter's Health

B1. Do you have any children? ☐ Yes ☐ No

B2. If yes, please list the age of each child, including adult children.

Age _______ ☐ Female ☐ Male
Age _______ ☐ Female ☐ Male
Age _______ ☐ Female ☐ Male
Age _______ ☐ Female ☐ Male
Age _______ ☐ Female ☐ Male
Age _______ ☐ Female ☐ Male
Age _______ ☐ Female ☐ Male

HPV (human papillomavirus) is a common sexually transmitted infection that sometimes leads to genital warts and cervical cancer. <u>HIV</u> infection increases your chances of developing cervical cancer. Cervical cancer affects the cervix, which is the opening to the womb. <u>Cervical cancer is the most common female cancer in Botswana.</u>

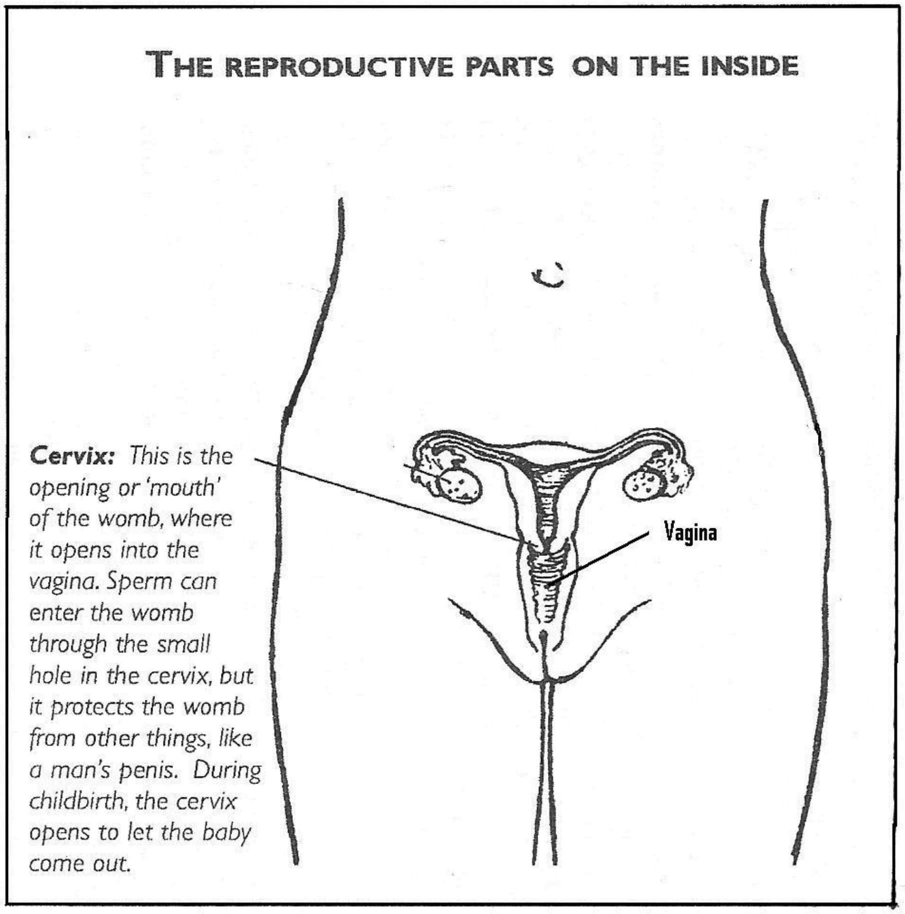

The next questions are about daughters aged 9-18. If you do not have a daughter, please answer these questions as **if you do.** If she is not ages 9-18 imagine her **being age 9-18**.

B3. What is the chance that your daughter will be infected with **human papillomavirus** or **HPV** in her lifetime?

☐ No chance ☐ Low ☐ Moderate ☐ High

B4. If your daughter were infected with **HPV**, how serious a threat to her health would it be?

☐ No threat ☐ Low ☐ Moderate ☐ High

B5. What is the chance that your daughter will get **<u>cervical cancer</u>** in the future?

☐ No chance ☐ Low ☐ Moderate ☐ High

B6. If she did get **<u>cervical cancer</u>**, how serious a threat to her health would it be?

☐ Extremely low ☐ Low ☐ Moderate ☐ High ☐ Extremely high

The next questions are about **HIV** and genital warts, both infections transmitted by sex.

B7. What is the chance that your daughter will be infected with **HIV** in her lifetime?

☐ No chance ☐ Low ☐ Moderate ☐ High ☐ She already has **HIV**

B8. If your daughter were infected with **HIV**, how serious a threat to her health would it be?

☐ Extremely low ☐ Low ☐ Moderate ☐ High ☐ Extremely high

B9. What is the chance that your daughter will be infected with genital warts in her lifetime?

☐ No chance ☐ Low ☐ Moderate ☐ High ☐ She already has genital warts

B10. If your daughter had genital warts, how serious a threat to her health would it be?

☐ Extremely low ☐ Low ☐ Moderate ☐ High ☐ Extremely high

C. HPV vaccine: An HPV vaccine is now available that protects against most genital warts and cervical cancer. Sometimes it's called the cervical cancer vaccine, HPV shot, Cervarix or Gardasil. We will call it the HPV vaccine.

C1. Have you heard of the HPV vaccine before today?

☐ Yes ☐ No

D. Vaccinating your Daughter: When the new HPV vaccine becomes available in Botswana, it will probably be recommended for girls between ages 9 and 18.

Please answer the next questions thinking about your daughter. If you do not have a daughter, please answer these questions **as if you do**. If she is not ages 9-18 imagine her **being age 9-18**.

	Definitely won't	Probably won't	Probably will	Definitely will
D1. How likely are you to get your daughter the HPV vaccine when it becomes available?	☐	☐	☐	☐
D2. If a doctor recommended the HPV vaccine for your daughter, would you give it to her?	☐	☐	☐	☐
D3. If a nurse recommended the HPV vaccine for your daughter, would you give it to her?	☐	☐	☐	☐
D4. Would you give the HPV vaccine to your daughter if it were available with other **childhood** vaccines?	☐	☐	☐	☐

	Strongly Disagree	Disagre e	Agree	Strongly Agree
D5. I don't have enough information about the HPV vaccine to decide whether to give it to my daughter.	☐	☐	☐	☐
D6. If my daughter gets the HPV vaccine, she may be more likely to have sex.	☐	☐	☐	☐

E. Imagine you WANT to get the HPV vaccine to for your daughter and it is available in Botswana.

E1. Where would you first go to get her the HPV vaccine? (please check ONLY one)

☐ Family doctor or general practitioner ☐ Pediatrician ☐ Gynecologist or OB/GYN
☐ Public or community clinic ☐ School clinic ☐ Other: ____________________

E2. If the HPV vaccine were available at your daughter's school, would you want her to get it there?
☐Yes ☐ No

E3. How hard do you think it would be to find a provider or clinic that is easy to get to for the HPV vaccine?

☐ Not hard at all ☐ Somewhat hard ☐ Hard ☐ Very hard

E4. Again, imagine the HPV vaccine is available here, list three reasons that would make getting the HPV vaccine for her difficult.

#1 (most difficult)____

#2 ____

#3 ____

E5. Please list all those who would make the decision to get the HPV vaccine for your daughter: (check all that apply)

☐ Me ☐ My spouse/partner ☐ My daughter ☐ An elder ☐ Her doctor
☐ Other______________________________

E6. How much would you discuss the decision with your daughter to get her the HPV vaccine?

☐ Not at all ☐ A little ☐ A moderate amount ☐ A lot

E7. If the HPV vaccine were **NOT** paid for by the government, what is the most you would pay of your own money to get the HPV shots for your daughter? ______________ pula

F. Pap smear: Screening for Cervical Cancer. The Pap test, also called a Pap smear, checks for changes in the cells of your cervix. The Pap test can tell if you have an infection, abnormal (unhealthy) cervical cells, or cervical cancer. How is a Pap test done? Your doctor takes a sample of cells from your cervix to be tested and examined. While you lie on an exam table, the doctor puts a special instrument called a speculum into your vagina, opening it to see the cervix. Your doctor will then use a special stick or brush to take a few cells from inside and around the cervix.

F1. Have you ever had a Pap test? ☐ Yes ☐ No ☐ N/A (because I am male)

F2. Are you willing to get a Pap test in this next year? ☐ Yes ☐ No ☐ N/A (because I am male)

F3. Please list the reason for your answer to F2. If you answered yes, why would you have a Pap test? If you answered no, why you would NOT get one done?

#1 ____

#2 ____

#3 ____

G. Background: **The next questions about your background will help us understand study participants. Your answers are completely anonymous and confidential.**

G1. Has someone you care about ever had cervical cancer?

☐ Yes ☐ No

G2. Has a doctor or other medical professional ever told you that you have **cervical cancer**?

☐ Yes ☐ No ☐ N/A (because I am male)

G3. Has a doctor or other medical professional ever told you that you have **HIV (human immuno-deficiency virus) or AIDS**?

☐ Yes ☐ No ☐ Never tested

G4. Has a doctor or other medical professional ever told you that you have genital warts?

☐ Yes ☐ No

G5. Has a doctor or other medical professional ever told you that you had other **sexually transmitted infections** like chlamydia, gonorrhea, herpes ulcers, or syphilis?

☐ Yes ☐ No

G6. What is your age? ________

G7. What is your marital status?

☐ Married or living as married
☐ Divorced
☐ Widowed
☐ Separated
☐ Never married

G8. What is the highest level of education you completed?

☐ Less than primary education
☐ Primary education
☐ Secondary education

Tertiary education:
☐ Certificate
☐ Diploma
☐ Bachelor's
☐ Masters or Doctorate

G9. What is your religious background? ___________________________________

G10. What town/city do you currently live?___________________________________

G11. Do you have a regular source of income? ☐ Yes ☐ No
If yes, what is your monthly income in pula
☐ Less than 1000 ☐ 1000-2499 ☐2500-4999 ☐5000-10000 ☐ More than 10000

Thank you for participating in the Gaborone Children's Health Study. What we learn from you and other parents may affect policies about HPV vaccination in Botswana and help girls in the country get better health care.

Tshekatsheko ya botsogo jwa bana ba Gaborone

Re leboga thuso ya gago mo dipatlisisong tse di botlhokwa tse e leng maiteko a boditshabatshaba. Potsolotso e ke ya dintlha dingwe tse di botlhokwa tsa botsogo tse di amang bomme le ba malwapa a bone. Re eletsa go itse maikutlo a gago ka dintlha tse di botlhokwa.

**A. General**	Ee	Nnyaa
A1. A o kile wa utlwa ka HPV?<br>HPV e farologana le mogare wa **HIV.**	☐	☐
A2. A o kile wa utlwa ka dikakana tsa ko bosading?	☐	☐
A3. A o kile wa utlwa ka kankere ya molomo wa popelo?	☐	☐fa karabo ele **nnyaa** feteela kwa go **B**

A4. Fa mosadi a na le kankere ya molomo wa popelo, bolela dilo dile tharo tse o akanyang di katswa di bakile bolwetse jo?

#1 (eka bakwa ke)____

#2 ____

#3____

Dipotso tse di latelang kaga **HIV** (human immunodefiecy virus) le ka kankere ya molomo wa popelo.

	**Seemo se ko tlase**	**Ga gona pharologanyo**	**Seemo se ko godimo**	**Ga ke itse**
A5. O akanya gore **HIV** e ama jang seemo Sa mosadi gore a nne le kankere ya molomo wa popelo?	☐	☐	☐	☐
A6. O akanya gore **HIV** e ama jang seemo sa mosadi gore a nne le mogare wa HPV?	☐	☐	☐	☐

B. Botsogo jwa ngwana wa Mosetsana

B1. A o na le bana? □ **Ee** □ **Nnyaa**

B2. Fa karabo ele ee, tswee tswee kwala dingwaga tsa ngwana mongwe le mongwe, o akaretse le ba batona.

Dingwaga_______________□ mosetsana □ mosimane
Dingwaga______________ □ mosetsana □ mosimane
Dingwaga_______________□ mosetsana □ mosimane
Dingwaga_______________□ mosetsana □ mosimane
Dingwaga_______________□ mosetsana □ mosimane
Dingwaga_______________□ mosetsana □ mosimane
Dingwaga_______________□ mosetsana □ mosimane

HPV (Human Papilloma Virus) ke mogare oo tlhwaelesegileng wa malwetse a dikobo o o ka bakang kankere ya molomo wa popelo le dikakana tsa bosadi/bonna nako tse dingwe. Mogare wa <u>HIV</u> o oketsa/atisa seemo sa go ka tsenwa ke malwetse aa bakwang ke mogare HPV le kankere ya molomo wa popelo tota. <u>Mo Botswana kankere ya mlomo wa popelo ke yone ee atigileng thata mo go bomme.</u>

Se ke molomo wa popelo. Peo ya senna e tsena mo popelong ka phatlhanyana ya molomo wa popelo,mme o thibele dilo dingwe jaaka bonna go tsena kana fetela kwa popelong. Ka nako ya pelegi molomo wa popelo oa bulege go letla lesea go tsholwa.

Bosadi(Vagina)

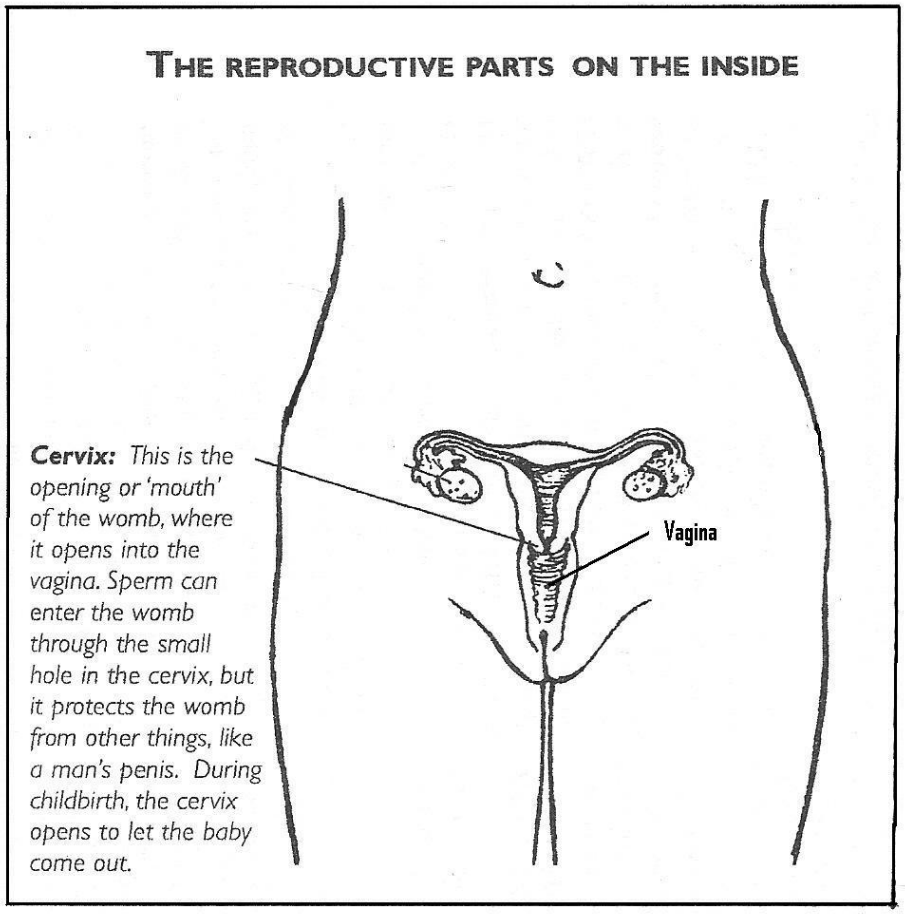

Dipotso tse di latelang ke ka bana ba gago ba basetsana. Fa o sena bana ba basetsana, **tswee tswee di arabe jaaka o kare o na le bone**. Fa ase dingwaga tse di magareng ga boferabognwe le lesome le boferabobedi **akanyetsa ale mo dingwageng tseo.**

B3. Kgonagalo ya gore ngwana/bana ba gago ba basetsana ba tsenwe ke **HPV** mo botshelong jwa bone e kae?

☐ Ga e yo ☐ E kwa tlase ☐ E fa gare ☐ E kwa godimo

B4. Fa ngwana/bana ba gago ba basetsana bane ba tsenwe ke **HPV**, go ka nna diphatsa go le kae mo go bone?

☐ Ga go diphatsa ☐ Kwa tlase ☐ Fa gare ☐ Kwa godimo

B5. Go na le kgonagalo e e kae gore ngwana/bana ba gago ba basetsana ba tsenwa ke kankere ya molomo wa popelo mo isagong?

☐ Ga e yo ☐ E kwa tlase ☐ E fa gare ☐ E kwa godimo

B6. Fa a/ba ka tsenwa ke kankere ya popelo, se se ka nna diphatsa go le kae mo botsogong jwa gagwe/bone?

☐ Kwa tlase thata ☐ Kwa tlase ☐ Fa gare ☐ Kwa godimo ☐ E kwa godimo thata

Dipotso tse di latelang ke ka HIV le Dikakana tsa ka fa tlase , ele malwetse a tlhakanelo dikobo.

B7. Kgonagalo ya gore ngwana/bana ba gago ba basetsana ba tsenwe ke mogare wa **HIV** mo botshelong jwa bone e kae?

□ Ga e yo □ E kwa tlase □ E fa gare □ E kwa godimo □ O setse ana le mogare wa **HIV**

B8. Fa bana/ngwana wa gago wa mosetsana a ka bo ane a tsenwe ke mogare wa **HIV**, se sene se ka nna diphatsa go le kae mo botsogong jwa gagwe?

□Kwa tlase thata □ Kwa tlase □Fa gare □Kwa godimo □E kwa godimo thata

B9. Kgonagalo ya gore ngwana/bana ba gago ba basetsana ba nne le dikakana tsa ko bosading mo botshelong jwa bone e kae?

□Ga e yo □E kwa tlase □E fa gare □E kwa godimo □O setse ana le dikakana

B10. Fa bana/ngwana wa gago wa mosetsana ane a na le dikakana tsa ko bosading se sene se ka nna diphatsa go le kae mo botsogong jwa gagwe?

□Kwa tlase thata □ Kwa tlase □Fa gare □Kwa godimo □E kwa godimo thata

C. Mokento wa HPV:

Mokento wa HPV o jaanong o teng o o sireletsang kgatlhanong le `dikakana tsa ko bosading (genital warts) le kankere ya molomo wa popelo. Fa gongwe o bidiwa mokento wa kankere ya molomo wa popelo, HPV shot, kgotsa Gardasil. Mme re tlaa o bitsa mokento wa HPV.

C1. A o kile wa utlwa ka mokento wa HPV pele ga letsatsi leno?
☐ Ee ☐ Nnya

D. Go fiwa mokento ga ngwana wa gago wa Mosetsana:

Fa mokento wa HPV oka nna teng mo Botswana, o ka letlelelwa go wa fiwa bana ba basetsana ba ba dingwaga di fa gare ga ferabongwe le lesome le boferabobedi.

Ka tsweetswee araba dipotso tse di latelang o akantse ngwana wa gago wa mosetsana. Fa o sena ngwana wa mosetsana araba jaaka o kare o na nae. Fa a se dingwaga tse di fa gare ga **9 le 18 mo** akanyetse ale mo dingwageng tseo.

	Gotlhelele ga ke kake	Gongwe ga ke kake	Gongwe ke tlaa dira	Ke tlaa dira
D1. Go na le kgonagalo e e kae gore o fe ngwana wa gago wa mosetsana mokento wa HPV fa o nna teng?	☐	☐	☐	☐
D2. Fa ngaka a ka go fa tetla ya go dirisa mokento wa HPV, a o ka o fa ngwana wa gago wa mosetsana?	☐	☐	☐	☐
D3. Fa mooki a ka go fa tetla ya go dirisa mokento wa HPV, a o ka o fa ngwana wa gago wa mosetsana?	☐	☐	☐	☐
D4. A o ka fa ngwana wa gago wa mosetsana mokento wa HPV fa o ne o fiwa le **mekento e mengwe ya bana?**	☐	☐	☐	☐

	Ga ke dumelane gotlhelele	Ga ke dumelane	Ke a dumelana	Ke dumelana thata
D5. Ga kena dikitso tse dintsi ka mokento wa HPV go tsaya tshwetso ya go o dirisa.	☐	☐	☐	☐
D6. Fa ngwanake wa mosetsana a ka fiwa mokento wa HPV, go na le kgonagalo e ntsi ya gore a tlhakanele dikobo.	☐	☐	☐	☐

E. Akanya o eletsa go fa ngwana wa gago wa mosetsana mokento wa HPV mme ebile ole teng mo Botswana.

E1. O ka ya kae pele? (**tsweetswee tshwaya gangwe fela**)

□ Ngaka ya lolwapa □ Ngaka ya bana □ Ngaka ya bomme

□ Kokelwana ya motse □ Kokelwana ya sekole. Tse □ Dingwe_________________

E2. Fa o ne o fiwa ko sekoleng, o ne o ka batla/eletsa ngwana wa gago gore a o fiwe teng?
□Ee □Nnyaa

E3. O akanya gore go tla go thatafelele go le kae go tsaya mokento ko kokelwaneng?
□ Ga go thata gotlhelele □ Gongwe go ka nna thata □ Go thata □Go thata mo go feteletseng

E4. Akanyetsa fa mokento wa HPV ole teng mo Botswana,jaanong bolela mabaka a le mararo a a ka go ketefaletsang go bona mokento o?

#1 (sese kago ketefaletsang thata)_____________________________________

#2 ____

#3 ____

E5. Tswee-tswee kwala batho ba o ka tsayang tshwetso le bone fa ngwana wa gago a ka fiwa mokento mo isagong: (Tshwaya **botlhe** ba ba ka kgonang)
□ Ke nna □ Ke mopati wame □ Ngwanake wa mosetsana □ Mongwe yo Mogolwane □ Ngaka ya gagwe □Mongwe_____________________

E6. O ka buisana go le kae le ngwana wa gago wa mosetsana ka tshwetso e?
□ Gotlhelele □ Go le go nnye □ Mo go lekanetseng □ Thata

E7. fa mokento wa HPV one o sa duelelwe ke goromente , o ne o ka duela bo kae mo mading a gago gore ngwana wa gago wa mosetsana a fiwe mokento o? P__________

<u>F. Pap smear:</u> Tlhatlhobelo ya Kankere ya molomo wa popelo.

Go tlhatlhobela kankere ya molomo wa popelo. Tlhatlho bo e, ke go bona gore a gona le ddiphetogo dingwe mo molomong wa popelo ya gago. Tlhatlhobo e, e ka go tlhalosetsa gore a ona le bolwetse, (ditshika tse di sa itekanelang mo molomong wa popelo kana kankere).
<u>Tlhatlhobo e e dirwa jang?</u> Ngaka o tsaya leswenyana mo molomong wa popelo go le tlhatlhoba. O robala mo bolaong jwa tlhatlhobo, go bo go tsenngwa tshitswana mo bosading go bula molomo wa popello. Ngaka o tla dirisa didirisiwa tsa tlhatlhobo (jaaka borashe) go tsaya matute mo tikologong ya molomo wa popelo. Matute a, a tshasiwe mo galaseng, a isiwe kwa lefelong la tlhatlhobo, go tlhatlhobiwa.

F1. Ao kile wa utlwa ka tlhobotlhobo ya kankere ya molomo wa popelo (pap smear?) ☐ **Ee** ☐ **Nnyaa** ☐ **N/A**

F2. Ao na le maikaeleo a go itlhatlhbela kankere ya molomo wa popelo dira pap smear ngawana ono? ☐ **Ee** ☐ **Nnyaa** ☐**N/A**

F3. Tsweetswee fa lebaka la karabo ya potso F2. Fa karabo ele ee, ke eng o ka itlhatlhobela kankere ya molomo wa popelo (pap smear) kana fa karabo ele nnyaa, ke eng o ka sa kake o e itlhatlhobele?

#1 ____

#2 ____

#3 ____

<u>G. Dikitso ka ga gago:</u> Dipotso tse di latelang ke ka dikitso ka ga gago di tlaa re thusa go itse batsenelela dipatlisiso. Dikarabo tsa gago mabapi le dipotso tse ga di kake tsa itsewe ke ope gape e tlaa nna sephiri.

G1. A mongwe yo o mo ratang o kile a amiwa ke kankere ya molomo wa popelo?
☐ Ee ☐ Nyaa

G2. A ngaka kgotsa mongwe wa badiri ba botsogo ba kile ba go bolelela gore o na le <u>kankere ya molomo wa popelo</u>?

☐ Ee ☐ Nyaa ☐ N/A

G3. A ngaka kgotsa mongwe wa badiri ba botsogo ba kile ba go bolelela gore o na le mogare wa **<u>HIV</u>** kgotsa bolwetse jwa **<u>AIDS</u>**?

☐ Ee ☐ Nyaa ☐ Gas ke ise ke itlhatlhobe

G4. A ngaka kgotsa mongwe wa badiri ba botsogo ba kile ba go bolelela gore o na le dikakana ka ko bosading?

☐ Ee ☐ Nyaa

G5. A ngaka kgotsa mongwe wa badiri ba botsogo ba kile ba go bolelela gore o na le malwetse a a anamang ka tlhakanelo dikobo jaaka, Chlamydia,dintho mo bosading (genetal /herpes ulcers) , rasephiphi kana thosola?

☐ Ee ☐ Nyaa

G6. O dingwaga di kae? ________

G7. Tshwaya seemo sa gago sa nyalo?

☐ Ke nyetswe
☐ Ke kgaogane le monna
☐ Ke tlhokafaletswe ke monna
☐ Ke kgaogane le monna lobaka lo lokhutshwane
☐ Ga ke ise ke nyalwe

G8. O tsene sekole go fitlha fa kae?

☐ Kwa tlase ga sekole se se botlana
☐ Sekole se se botlana
☐ Sekole se segolwane
☐ Mmadikolo
☐ Sekole sa dithuto tse dikgoo
☐ Dithuto tse di tseneletseng (Masters, PhD, MD,JD)

G9. Tumelo ya gago ke efe?___________________________________

G10. O nna mo toropong kana motse ofe?___________________

G11. A o na le tuelo e o e bonang kgwedi le kgwedi? ☐ Ee ☐ Nyaa
Fa go le jalo, tuelo e ke bokae kgwedi le kgwedi:
□ Kwa tlase ga 1000 □ Fa gare ga 1000 le 2499 □ Fa gare ga 2500 le 4999
□ Fa gare ga 5000 le 10000 □ Kwa godimo ga 10000

Re leboga gore o bo tsere karolo mo dipatlisisong tsa boditshatshaba ka botsogo jwa bana. Se re tlaa se ithutang mo go wena le batsadi ba bangwe se ka nna sa ama ditsamaiso ka mokento wa HPV mo Botswana, mme gape se thuse bana ba basetsana mo lefatsheng go bona ditlamelo tsa botsogo tse di botoka
